# Diverse Strategies Used by Picornaviruses to Escape Host RNA Decay Pathways

**DOI:** 10.3390/v8120335

**Published:** 2016-12-20

**Authors:** Wendy Ullmer, Bert L. Semler

**Affiliations:** Department of Microbiology and Molecular Genetics, School of Medicine, University of California, Irvine, CA 92697, USA; wullmer@uci.edu

**Keywords:** picornavirus, *Picornaviridae*, poliovirus, coxsackievirus, human rhinovirus, RNA degradation, mRNA decay, RNA stability, RNase L, deadenylase

## Abstract

To successfully replicate, viruses protect their genomic material from degradation by the host cell. RNA viruses must contend with numerous destabilizing host cell processes including mRNA decay pathways and viral RNA (vRNA) degradation resulting from the antiviral response. Members of the *Picornaviridae* family of small RNA viruses have evolved numerous diverse strategies to evade RNA decay, including incorporation of stabilizing elements into vRNA and re-purposing host stability factors. Viral proteins are deployed to disrupt and inhibit components of the decay machinery and to redirect decay machinery to the advantage of the virus. This review summarizes documented interactions of picornaviruses with cellular RNA decay pathways and processes.

## 1. Introduction

Cytoplasmic RNA viruses encounter a myriad of host defense mechanisms that must be countered by a small arsenal of viral proteins. Preserving the stability and integrity of viral RNA (vRNA) is of fundamental importance to the virus to ensure successful generation of progeny virions. Throughout the virus replication cycle, vRNAs encounter multiple potentially destabilizing host cell pathways and processes, from regulated mRNA decay pathways to interferon (IFN)-induced vRNA decay. Members of the *Picornaviridae* family are small, positive-sense single-stranded RNA viruses that have evolved strategies to re-purpose, inhibit, or otherwise evade many components of the cellular RNA decay machinery.

As a family, picornaviruses are composed of at least 29 different genera which include many significant human and animal pathogens causing a range of illnesses and economic burden. The Enterovirus genus of picornaviruses includes the causative agents of paralytic poliomyelitis (poliovirus), hand, foot, and mouth disease (coxsackievirus (CVA) A16 and enterovirus (EV) 71), and the common cold (human rhinovirus (HRV)). Other severe symptoms of EV infection include meningitis, encephalitis, myocarditis, and pericarditis, which can arise from infection by subtypes of CVA, coxsackievirus B (CVB), EV, or echovirus. The lone member of the Hepatovirus genus, hepatitis A virus (HAV), infects the liver and, in rare cases, causes acute liver failure. The Cardiovirus genus includes encephalomyocarditis virus (EMCV), Theiler’s murine encephalomyelitis virus (TMEV), and Saffold virus (SAFV). EMCV and TMEV are largely non-human pathogens that cause symptoms that include myocarditis, encephalitis, and, for EMCV specifically, reproductive failure in pigs. SAFV is a recently discovered human cardiovirus that does not yet have clearly defined pathological features but has been linked to acute flaccid paralysis, meningitis, and cerebellitis. Foot-and-mouth disease virus (FMDV), a member of the Aphthovirus genus, is one of the most economically important livestock viruses. Infection by FMDV causes painful vesicles in the feet, mouth, and teats of cloven-hoofed animals, reducing their productivity and requiring significant eradication measures.

Picornaviruses induce extensive modification of cellular processes to complete their replication cycle. Upon attachment and release of genomic RNA into the cytoplasm of a host cell, viral proteins are translated by a cap-independent mechanism using an internal ribosome entry site (IRES) located in the 5′ non-coding region (5′ NCR) of the vRNA. Picornavirus genomic RNAs are linked to a small, virus-derived protein at their 5′ termini (VPg), possess a 3′ poly(A) tract, and encode a single open reading frame that is translated into one polyprotein. Viral proteinases process the viral polyprotein into precursor and mature proteins, resulting in the formation of four structural proteins and seven to eight mature non-structural proteins, depending on the virus. Viral proteins induce membrane rearrangements to form replication complexes, which are sites for RNA synthesis by the vRNA-dependent RNA polymerase, 3D. Newly synthesized RNAs undergo further rounds of translation/replication or become packaged into progeny virions. To redirect host resources toward virus replication, many picornaviruses rapidly shut down cap-dependent translation and disrupt nucleocytoplasmic trafficking to relocalize nuclear factors required for replication into the cytoplasm [1]. Modification of the cellular landscape is largely accomplished through the actions of non-structural proteins, particularly the viral proteinases 2A, 3C, and L (L is encoded by FMDV) [2,3].

**Figure 1:**
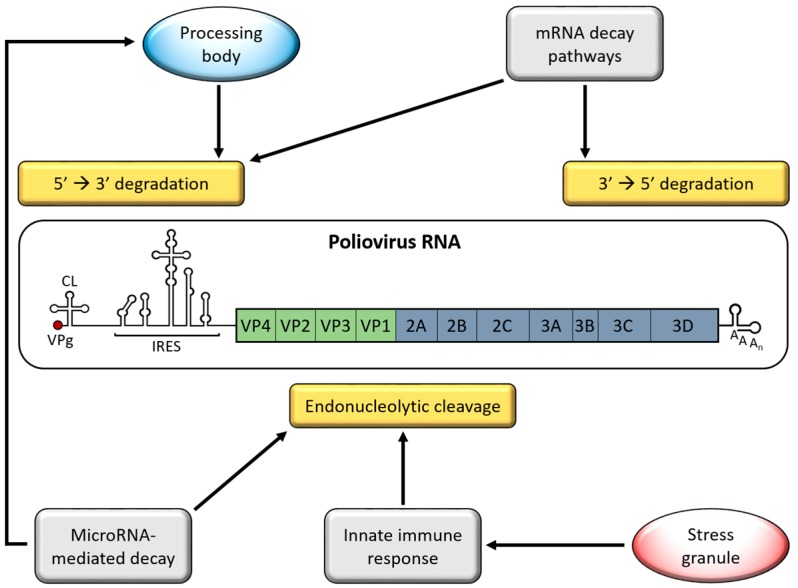
Poliovirus interactions with host RNA decay pathways. Picornavirus RNAs are exposed to degradation machinery from multiple RNA decay pathways and processes including 5′→3′ degradation in processing bodies (PBs), 5′→3′ or 3′→5′ degradation by mRNA decay pathways, and endonucleolytic cleavage as a result of activation of the innate immune response or microRNA-mediated decay. Poliovirus RNA serves as a model for the picornavirus genome. Genomic RNA is linked to a small virus-encoded protein, VPg, at the 5′ terminus. The 5′ non-coding region (NCR) contains a cloverleaf structure (CL) and an internal ribosome entry site (IRES). A single open reading frame codes both structural (VP1-4) and non-structural (2A-3D) proteins that are proteolytically processed into precursor and mature viral proteins. The 3′ end of the genome encodes a 3′ NCR and poly(A) tract.

Picornaviruses encounter multiple cellular processes that destabilize vRNA (Figure 1). Given their cytoplasmic replication cycle, picornaviruses avoid nuclear RNA surveillance mechanisms but instead are susceptible to mRNA decay pathways that function in the cytoplasm, such as adenylate uridylate-rich element (ARE)-mediated mRNA decay (AMD). Generally, mRNA decay is initiated by deadenylation of the targeted transcript, followed by 5′→3′ or 3′→5′ degradation of the body of the mRNA by exonucleases. To ensure that vRNAs are not targeted by mRNA decay machinery, picornaviruses disrupt these processes at multiple levels. The strategies that picornaviruses employ to disrupt this antiviral response have been researched more extensively than picornavirus involvement in mRNA decay pathways. This review highlights research focused on picornavirus interactions with pathways or processes associated with RNA decay, largely focusing on EVs, for which the most experimental evidence exists.

## 2. Genome Stabilizing Features for Picornaviruses

Picornavirus RNAs have acquired several stabilizing features as a form of protection from cellular RNA decay machinery. At the 5′ end of the genome, the first barrier to decay machinery is a small, virus-encoded protein covalently bound to the 5′ terminal nucleotide of the vRNA, called VPg (Viral Protein, genome-linked) [4,5]. VPg is used by the vRNA polymerase, 3D, as a protein primer for RNA synthesis, resulting in vRNAs linked to VPg instead of a 7-methylguanosine (m7G) mRNA cap. Lacking a m7G cap, picornavirus RNAs are protected from cellular decapping enzymes like Dcp1 and Dcp2, whose activity initiates 5′→3′ RNA degradation. While cellular decapping enzymes are unable to hydrolyze the VPg-RNA bond, a cellular 5′-tyrosyl-DNA phosphodiesterase, TDP2, is capable of cleaving this bond and “unlinking” VPg from vRNA [6]. Cleavage of VPg may not result in destabilization of vRNAs since unlinked vRNA has been found to be associated with actively translating ribosomes and therefore protected from degradation, although the stability of unlinked vRNA has not yet been measured [7,8,9]. As an additional protection, the major 5′→3′ exonuclease, Xrn1, is degraded during poliovirus infection, preventing 5′→3′ digestion of unlinked vRNAs [10] (discussed further in Section 5).

For some picornaviruses, vRNA can inhibit endonucleolytic cleavage directly through association with ribonuclease L, (RNase L). RNase L activity is stimulated by the IFN response and contributes to the cellular defense against infection by degrading vRNA [11] (discussed further in Section 3). The antiviral activity of RNase L has been demonstrated for picornaviruses including EMCV and CVB4 [12,13]. However, poliovirus RNA is resistant to cleavage by RNase L through a structured RNA element located in the 3C proteinase coding sequence. This structured RNA binds the endoribonuclease domain of RNase L, which inhibits its activity [14]. This RNA element is conserved in group C EVs, which include poliovirus and several types of CVA, among others [15,16]. The element is not present in group A, B, or D EVs, of which CVB3 is a member and was found to be sensitive to RNase L. The protection of this RNA element from RNase L activity is not complete, however, as it was discovered that RNase L is still capable of cleaving the poliovirus genome at distinct locations [17].

Picornavirus RNA is stabilized through association with specific host proteins. Poly(rC)-binding protein 2 (PCBP2), an RNA-binding protein involved in mRNA stability and translation, binds 5′ stem-loop structures in poliovirus RNA to promote genomic RNA stability, viral translation, and RNA replication [18,19,20]. Mutation of one of these stem-loop structures to prevent PCBP2 binding results in diminished ability to form polysomes on poliovirus RNA, rendering the RNA susceptible to degradation [21,22]. PCBP2 also binds the 5′ NCR of CVB3, EV71 and HRV RNA to promote viral translation and RNA replication, but it has not yet been determined if this interaction affects vRNA stability [23,24,25,26]. Human antigen R (HuR) is a well-characterized mRNA stabilizing protein that was recently found to bind the EV71 5′ NCR and act as a positive regulator of translation [27]. While the effect of HuR on vRNA stability has yet to be determined, HuR may have a similar stabilizing effect as PCBP2, whereby promoting translation of vRNA protects it from degradation. HuR was previously shown to stabilize genomic RNAs of togaviruses, another family of positive-sense RNA viruses [28]. HuR was also identified as a poliovirus RNA-binding protein using thiouracil cross-linking mass spectrometry (TUX-MS), suggesting that it may have a similar effect on other vRNAs [29]. Host factors that promote picornavirus RNA stability have not been well studied, but it is likely that many of the proteins re-purposed for translation and replication serve a dual purpose in promoting vRNA stability.

## 3. Interferon (IFN)-Induced Viral RNA (vRNA) Degradation

The earliest defense against virus infection of cells involves activation of the innate immune response, which results in expression of genes that interfere with virus replication, prevent spread to neighboring cells, and trigger the adaptive immune response. Briefly described, detection of pathogen-associated molecular patterns (PAMPs) by cellular pattern recognition receptors (PRRs) initiates the innate immune response. During picornavirus infections, double-stranded RNAs (dsRNAs) that form during vRNA replication serve as the PAMP that is recognized by a PRR. PRR bound to viral dsRNA transduces the signal that a viral pathogen has been detected through multiple pathways, leading to the activation of transcription factors which promote the expression of IFN-β. IFN-β production ultimately results in transcription of hundreds of IFN-stimulated genes (ISGs) which collectively contribute to an antiviral state [30,31].

The IFN response activates multiple pathways to inhibit virus replication, including degradation of vRNA by RNase L. RNase L is normally expressed in mammalian cells, but remains inactive until infection is detected, resulting in ISG expression. Oligoadenylate synthetase (OAS) is an ISG that activates RNase L by generating 2′-5′ oligoadenylates (2-5A), the secondary messenger that induces dimerization and activation of RNase L [11,32]. Some picornaviruses have evolved mechanisms to directly inhibit RNase L activity. As noted above, RNase L can be directly inhibited by binding to a structured element within the RNA of group C EVs. Additionally, the TMEV L* protein has been shown to bind and inhibit RNase L [33]. The L* protein is an alternative, smaller form of L generated by leaky ribosome scanning [34]. In an uninfected cell, RNase L is inhibited by the cellular protein known as RNase L inhibitor/ATP-binding cassette, sub-family E member 1 (RLI/ABCE). EMCV infection induces RLI/ABCE expression, which contributes to RNase L inhibition [35]. It is not known whether other picornaviruses induce RLI/ABCE expression as well.

Several strategies are employed by picornaviruses to prevent endonucleolytic cleavage by RNase L prior to the nuclease becoming active. Melanoma differentiation-associated gene 5 (MDA5) is the PRR responsible for detecting the replicative form of picornavirus RNAs [36,37,38,39,40,41,42]. To avoid detection, MDA5 is cleaved or degraded during infection by poliovirus, CVB3, EV71, EMCV, or HRV1a. For poliovirus, CVB3, and EV71, the 2A proteinase appears to be responsible for cleaving MDA5; however, conflicting reports also indicate that cleavage may occur in a proteasome- or caspase-dependent manner [39,43,44]. Interestingly, MDA5 degradation is not common to all picornavirus infections, as MDA5 remains intact during HRV16 or echovirus type 1 infection [44]. Ligand-bound MDA5 assembles with the adaptor molecule mitochondrial antiviral signaling protein (MAVS) at the mitochondrial membrane to transfer the signal downstream. Accordingly, MAVS is also targeted for inhibition during viral infection. MAVS proteolysis has been observed during infection by poliovirus, CVB3, EV71, HRV1a, or HAV [43,45,46,47]. Several lines of evidence point toward MAVS cleavage by the 2A and/or 3C proteinases, as well as by caspases. In one study, expression of poliovirus, CVB3, or EV71 2A proteinase alone resulted in MDA5 and MAVS cleavage similar to what is observed during infection [43]. FMDV infection also results in reduction of MAVS, but not through its cleavage. Instead, the non-structural protein, 3A, and structural protein, VP3, have both been shown to down-regulate MAVS mRNA expression [48,49].

IFN-β expression is induced following recruitment of signaling molecules to MAVS which activates TANK-binding kinase 1 (TBK1), resulting in phosphorylation of the transcription factors IFN regulatory factor 3 and 7 (IRF-3 and -7). Phosphorylated IRF-3/7 proteins translocate to the nucleus to activate transcription of IFN-β. EV71 3C proteinase cleaves IRF-7, inhibiting its ability to transactivate IFN-β expression [50]. FMDV employs a different strategy to inhibit IFN-β expression. The FMDV Lb protein (generated by leaky ribosome scanning, similar to L*) deubiquitinates TBK1 and the signaling molecule TNF receptor-associated factor 3 (TRAF3), thereby inhibiting their activity [51]. In addition, the FMDV L protein causes a decrease in IRF-3/7 mRNA levels [52].

MAVS also activates proinflammatory cytokine expression by the nuclear factor-κB (NFκB) transcription factor. NFκB activation requires phosphorylation of the NFκB inhibitor-α (IκBα) by the IκB kinase complex, IKK (composed of IKKα, IKKβ, IKKγ). Phosphorylation of IκBα releases NFκB, allowing it to translocate to the nucleus and activate transcription. Various strategies are employed by picornaviruses to inhibit NFκB activation. Poliovirus, CVA16, CVB3 and EV71 2C proteins have been shown to inhibit phosphorylation and activation of IKK by recruiting protein phosphatase 1 (PP1) to IKKβ [53,54]. FMDV inhibits IKK through cleavage of IKKγ by 3C proteinase [55]. NFκB is also targeted directly through its p65/RelA subunit. Poliovirus, HRV14, echovirus type 1, and FMDV cleave p65/RelA during infection [56,57] and the EV71 2C protein has also been shown to inhibit NFκB by binding to p65/RelA [58].

**Figure 2:**
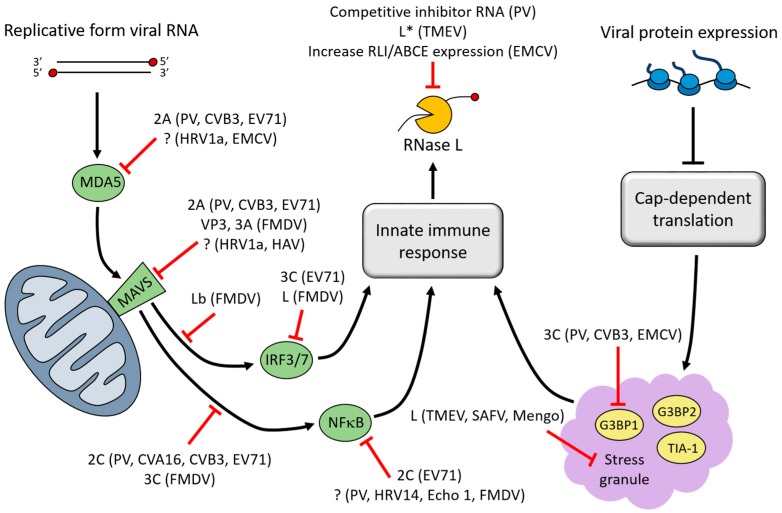
Inhibition of ribonuclease L (RNase L) activation by picornavirus proteins. RNase L is an effector molecule of the innate immune response. In its active form, RNase L endonucleolytically cleaves viral RNA (vRNA). Several picornaviruses have been shown to inhibit RNase L directly through binding of a viral inhibitor to RNase L or up-regulation of the cellular RNase L inhibitor, RLI/ABCE. Picornaviruses also indirectly prevent activation of RNase L through extensive disruption of pathways that contribute to the innate immune response. The signaling cascade that is initiated by MDA5 following detection of viral double-stranded RNA is disrupted at multiple steps during infection, which prevents expression of interferon (IFN)-induced genes and activation of RNase L. Additionally, the disassembly of stress granules (SGs) during infection inhibits SG-mediated enhancement of the innate immune response. Simplified pathways are depicted here, highlighting points in the pathway that are inhibited by specific picornaviruses and the viral protein responsible (“?” indicates that the viral protein responsible is unknown). PV, poliovirus; CVA16, coxsackievirus A16; CVB3, coxsackievirus B3; EV71, enterovirus 71; Echo 1, echovirus type 1; HRV1a, human rhinovirus 1a; EMCV, encephalomyocarditis virus; Mengo, mengovirus (a strain of EMCV); TMEV, Theiler’s murine encephalomyelitis virus; SAFV, Saffold virus; HAV, hepatitis A virus; FMDV, foot-and-mouth disease virus.

Picornaviruses antagonize the innate immune response at many steps in the pathway, including steps not mentioned here (Figure 2). From preventing detection by vRNA sensors to inhibition and degradation of signal transduction molecules and transcription factors, picornaviruses employ a number of strategies to inhibit the antiviral response. Inhibition of this response promotes picornavirus replication and spread, in part by preventing the activation of RNase L, which poses a significant threat to vRNA stability.

## 4. Stress Granules (SGs)

Stress granules (SGs) are a type of cytoplasmic RNA granule that contain non-translating mRNAs and form in response to cellular stress and inhibition of translation [59,60,61]. Unlike processing bodies (PBs), which are RNA granules enriched for mRNA decay proteins, SGs contain many translation initiation factors and form around stalled translation initiation complexes [60,62,63,64]. SGs can assemble and disassemble continuously and may act as sites for mRNA storage and sorting between a repressed state, active translation on polysomes, or degradation in PBs [65,66,67]. Several mRNA decay factors associate with SGs, including the Xrn1 exonuclease [63], PMR1 endonuclease [68], and the mRNA decay proteins tristetraprolin (TTP), butyrate response factor 1 (BRF-1), and K homology-type splicing-regulatory protein (KSRP, also known as FBP2) [69,70]; however, SGs likely do not contribute to mRNA decay directly, but instead they promote degradation through their interaction with PBs. SGs and PBs can physically associate, share several protein components, and have been proposed to exchange “cargo,” thereby targeting translationally repressed mRNAs for degradation [63,71]. Conversely, SGs are also thought to stabilize non-translating mRNAs by temporarily sequestering them away from components of the decay machinery [72,73]. In the context of viral infection, the major contribution to the destabilization of vRNA by SGs may be through enhancement of the innate immune response.

Several picornaviruses have been shown to transiently induce the formation of SGs early in infection, which disperse at roughly middle to late times during infection. The transient induction of SGs has been visualized by immunofluorescence for the EVs poliovirus [60,74,75], CVB3 [76], and EV71 [77], and cardioviruses EMCV [78] and TMEV [79]. During poliovirus infection, some virus-induced SGs are compositionally unique from stress-induced SGs, containing the splicing factor and viral IRES transacting factor (ITAF), SRSF3 (SRp20) [80]. A driving force behind picornavirus-induced SG formation is the shut-down of host cap-dependent translation. This leads to the accumulation of stalled translation initiation complexes which induce the aggregation of SGs. For that reason, expression of poliovirus, CVB3, or EV71 2A proteinases alone, which cleave the cap-binding complex component eIF4G, can induce SG formation [76,77,81].

Assembly of SGs is mediated by the SG-nucleating proteins Ras-Gap SH3 domain-binding protein 1 and 2 (G3BP1 and G3BP2) and T-cell-restricted intracellular antigen 1 (TIA-1), among others [82,83,84]. For poliovirus, CVB3, and EMCV, the disassembly of SGs occurs as a result of G3BP1 cleavage by 3C proteinase [74,76,78]. Expression of uncleavable G3BP1 prevents the disassembly of SGs during infection, highlighting the importance of 3C cleavage in disrupting virus-induced SGs [76,78]. TIA-1 and G3BP2 remain intact during infection, although that does not exclude cleavage of other SG-nucleating proteins from contributing to the disruption of SGs. Interestingly, G3BP1 cleavage does not contribute to the disassembly of SGs formed following infection by TMEV, which is in contrast to EMCV. Instead, TMEV-induced SGs are inhibited by the L protein through an unknown mechanism. The SAFV and mengovirus L proteins are also capable of inhibiting SGs when expressed in place of TMEV L [79]. Other viral proteins may also contribute to the inhibition of SGs. Expression of the poliovirus structural protein-coding region P1, 2A proteinase, or 3A alone modestly inhibited SGs induced by oxidative stress, although it is not understood whether these proteins play a part in inhibition of virus-induced SGs [81].

Picornavirus-induced SGs have previously been implicated in activation of the IFN response, but until recently it was not known how direct activation occurs. SGs induced by influenza A virus infection, a (−) ssRNA virus from the *Orthomyxoviridae* family, contain vRNA and several antiviral proteins including the vRNA sensors RIG-I and MDA5, along with OAS and RNase L. Formation of these antiviral SGs parallel, and potentially activate, the IFN response and concentrate vRNA in proximity to RNase L [85]. Similarly, transient SGs formed during EMCV infection were also associated with an antiviral effect. Expression of uncleavable G3BP1 prior to infection, which prevents the disassembly of virus-induced SGs, resulted in significantly increased levels of IFN-β and other cytokines, as well as decreased virus replication [78]. SGs induced by the overexpression of G3BP1 inhibit the replication of CVB3, CVB5, and EV70 and contain proteins involved in the innate immune response, such as OAS2, RNase L, and double-stranded RNA-dependent protein kinase (PKR) [86]. These data indicate that a link exists between SGs, G3BP1, and the innate immune response.

Recent investigations have revealed that G3BP1 directly stimulates the antiviral response through recruitment of PKR to SGs. It was shown that G3BP1 directly binds PKR in SGs during mengovirus infection, and in complex with another SG nucleating protein, Caprin1, activates PKR [87]. Active PKR then moves into the cytoplasm, where it can mediate the innate immune response through both its kinase activity and role as an adaptor protein. PKR induces IFN expression through activation of the transcription factor NFκB. PKR activation of NFκB has been shown to occur through indirect phosphorylation of the NFκB inhibitor IκB, which results in IκB degradation and the translocation of NFκB to the nucleus [88,89]. Interestingly, while PKR is required for IFN-α/β induction during EMCV and TMEV infection, it does not appear to do so through transcriptional activation, but instead through regulation of IFN mRNA integrity. EMCV infection of PKR^−/−^ mouse cells resulted in very little IFN-β protein production despite normal IFN-β mRNA levels. Even though IFN-β mRNA levels appeared normal, the mRNA lacked a poly(A) tract, which resulted in its decreased translation [90].

Given a similar localization of PKR to EV-induced SGs, it would be reasonable to assume that PKR is activated in SGs formed by other picornaviruses, thereby enhancing the IFN response to infection. Even though RNase L was identified as a component of SGs formed following infection by CVB3, CVB5, or EV70, it is unlikely that this localization adds to the degradation of vRNA [87]. The proximity of RNase L to influenza A virus RNA in SGs was suggested as a possible mechanism for inhibition of virus replication; however, picornavirus RNA has not been detected in SGs, which has been tested for poliovirus, CVB3, and TMEV [75,76,79]. Therefore, any contribution of virus-induced SGs to vRNA decay can most likely be attributed to the induction of IFNs, which results in activation of RNase L and degradation of vRNA. The disassembly of SGs during picornavirus infection plays an important part of maintaining vRNA stability through suppression of IFN-induced RNA decay (Figure 2).

## 5. Processing Bodies (PBs)

PBs are constitutively expressed cytoplasmic RNA granules which are composed of non-translating mRNAs and many proteins, including those involved in mRNA decay such as decapping components (Dcp1, Dcp2, Lsm1-7), 5′→3′ exonuclease (Xrn1), and deadenylation factors (Pan2, Pan3, Ccr4, Caf1, poly(A) RNase (PARN)) [63,91,92,93,94]. Due to the concentration of mRNA decay machinery in PBs, these granules have been proposed to be involved in 5′→3′ mRNA decay [93,95]. However, nonsense-mediated decay (NMD) and AMD have both been shown to occur in the absence of PB formation despite localization of NMD or AMD proteins in PBs [70,96]. This suggests that PBs are not required for all types of mRNA decay, but may form as a consequence of mRNA degradation or silencing and serve to enhance the process [97]. Not all mRNAs that enter PBs are degraded. mRNAs targeted for miRNA-mediated translational repression can localize to PBs [98,99,100]. Upon relief of repression, these mRNAs may leave the PB and re-enter active translation [101,102]. Given their complex role in mRNA decay and storage, it is not surprising that picornaviruses have evolved ways of disrupting PBs to prevent vRNAs from aggregating in these granules.

The effect of picornavirus infection on PBs has been studied for two EVs, poliovirus and CVB3. Following infection by poliovirus or CVB3, PB foci were almost completely absent by mid-infection. The PB proteins Dcp1a and Xrn1 are degraded during poliovirus infection. Loss of Dcp1a likely occurs as a result of poliovirus 3C cleavage, while Xrn1 does not appear to be a target of either viral proteinase and is instead degraded through a proteasome-dependent pathway [10]. Degradation of Xrn1 may have an additional benefit in protecting non-VPg-linked vRNAs from digestion by this major 5′→3′ exonuclease. While cleavage and degradation of Dcp1a and Xrn1 likely contribute to PB disruption, the near-complete dispersal of PBs suggests that these granules are targeted through multiple mechanisms.

Deadenylation of mRNA is a necessary first step for PB formation. Inhibition of active deadenylation through the expression of dominant negative Caf1 results in almost complete disruption of PBs. Additionally, components of the deadenylase complexes Pan2–Pan3 and Ccr4–Caf1 localize to PBs, and siRNA-mediated knockdown of either Caf1, Ccr4, or Pan3 disrupt PB formation [94,103]. Therefore, inhibition of deadenylation presents an additional opportunity for virus-mediated disruption of PBs. Upon examination of deadenylase components following poliovirus infection, it was discovered that Pan3 is degraded, possibly as result of 3C cleavage, while PARN, Pan2, Ccr4, and Caf1 remain intact [10]. Knockdown of Pan3 has been shown to block PB formation but does not impair deadenylation of mRNAs [94]. Thus, poliovirus-mediated degradation of Pan3 may contribute to the disruption of PBs by preventing the association or nucleation of PB components, not through blocking deadenylation.

**Figure 3:**
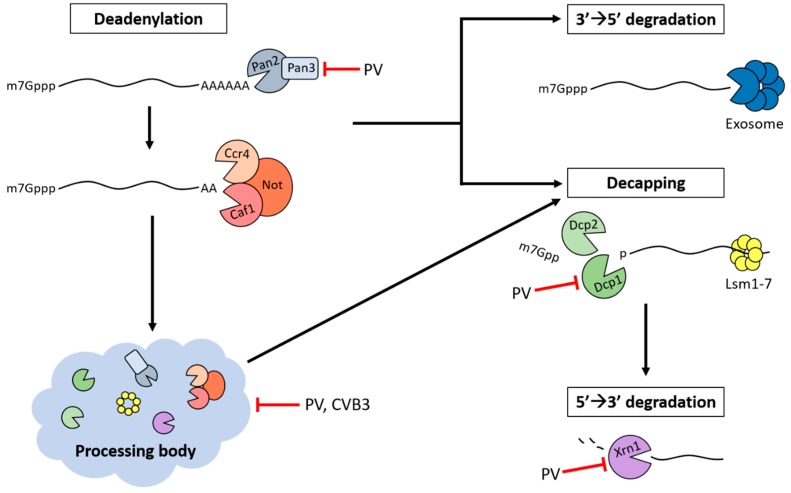
Inhibition of PBs and mRNA decay proteins during picornavirus infection. Deadenylation, decapping, and 5′→3′ RNA degradation machinery are targeted during poliovirus (PV) and coxsackievirus B3 (CVB3) infection. The localization of non-translating RNAs and mRNA decay proteins in PBs indicate that these RNA granules may be involved in 5′→3′ mRNA decay. PBs are dispersed during poliovirus and CVB3 infection, thereby disrupting their possible contribution to vRNA degradation. During poliovirus infection, Pan3 and Dcp1a are cleaved by 3C proteinase and Xrn1 is degraded by a proteasome-dependent mechanism.

Several poliovirus proteins promote the dispersal of PBs when expressed individually, although the mechanisms for disruption remain unclear. Expression of either the 2A or 3C proteinases alone significantly reduced the number of PBs formed per cell, with 2A having a more pronounced effect than 3C. Additionally, expression of either 3CD (a precursor of 3C that also possesses proteinase activity) or the vRNA polymerase, 3D, induced a modest reduction in PBs, although their molecular targets are unknown. The dispersal of PBs by 2A or 3C appears to occur through different mechanisms. Expression of 2A could neither prevent the formation of stress-induced PBs nor disrupt PBs containing exogenously expressed Dcp1a, while 3C could do both [81]. These data emphasize the importance of the disassembly and prevention of formation of PBs during poliovirus and CVB3 infection, a process that likely extends to other picornaviruses. The disruption of PBs highlights the significance of this process in the virus life cycle (Figure 3).

## 6. Adenylate Uridylate-Rich Element (ARE)-Mediated mRNA Decay (AMD)

Regulation of mRNA stability and turnover is a critical component of post-transcriptional gene expression. Both *cis*- and *trans*-acting elements participate in regulating the stability of mRNA. Many mRNAs encode AREs, which are sequences often found within the 3′ UTR that regulate mRNA stability through their association with the approximately 20 identified ARE-binding proteins (AUBPs). Stabilization or degradation of a transcript depends on which AUBPs are bound. Several AUBPs have been well characterized for promoting AMD of target transcripts: ARE/poly(U)-binding/degradation factor 1 (AUF1), KSRP, TTP, and BRF-1/2. Other AUBPs are better known for either stabilizing (HuR, HuD) or repressing the translation (TIA-1, TIAR) of target transcripts [104].

AMD occurs in the cell cytoplasm and is initiated by deadenylase digestion of the 3′ poly(A) tract, followed by degradation of the body of the mRNA using both 3′→5′ and 5′→3′ exonucleolytic pathways [105,106,107,108]. Several decay-promoting AUBPs have been shown to directly interact with components of the RNA degradation machinery, suggesting a mechanism for their initiation of mRNA decay [109,110]. It seems plausible that binding of certain AUBPs to vRNA could lead to recruitment of RNA degradation machinery and subsequent decay of vRNA. If so, then AMD could present an additional antiviral strategy utilized by the cell. To date, two AUBPs known for promoting AMD, AUF1 and KSRP, have been characterized for their roles in the picornavirus life cycle. Both proteins are shown to have a negative impact on virus replication, but apparently not through their expected functions in promoting RNA decay.

AUF1 (also known as hnRNP D) is one of the best-described AUBPs involved in AMD and has been shown to promote the decay of mRNAs encoding oncogenic, inflammatory, and cell cycle proteins, among others [111,112,113,114]. In addition to its role in promoting mRNA decay, AUF1 has also been shown to stabilize [115,116] and promote the translation of targeted transcripts [117]. AUF1 is expressed as four different isoforms generated through alternative pre-mRNA splicing, named p37, p40, p42, and p45 based on their apparent molecular weights [118]. All of the AUF1 protein isoforms are composed of the same two, non-identical RNA recognition motifs (RRMs) and a glutamine-rich domain, but display different affinities for ARE substrates and subcellular localization [119,120]. The two largest isoforms of AUF1, p42 and p45, localize primarily to the nucleus, while the smaller p37 and p40 isoforms are predominantly nuclear, but transit between the nucleus and cytoplasm [121].

A large-scale identification of AUF1 target transcripts using photoactivatable ribonucleoside-enhanced crosslinking and immunoprecipitation (PAR-CLIP) revealed that AUF1 isoforms bind over 3000 mRNAs as well as many non-coding RNAs (ncRNAs) [122]. Pairing PAR-CLIP with RNA-seq and polysome analysis following AUF1 overexpression implicated AUF1 in a range of regulatory events including stabilization and destabilization of both mRNA and ncRNA, promotion and repression of translation, and modulation of alternative splicing. In addition, AUF1 has recently been shown to have a role in the life cycle of DNA and RNA viruses including Epstein-Barr virus [123,124], HIV [125], hepatitis C virus [126], West Nile virus [127,128], and several picornaviruses [129,130,131,132].

A direct interaction between AUF1 and picornavirus RNA was originally discovered from an RNA affinity screen using the 5′ NCR of poliovirus RNA [133]. This finding was later expanded to include binding to HRV, CVB3, and EV71 RNA [129,132,133]. It was subsequently discovered that AUF1 knockout or knockdown in human or mouse cells resulted in increased replication of these viruses, suggesting that AUF1 may act as a host restriction factor to EV infection [129,130,131,132]. Prior to these discoveries, a study of HRV-16 infection of human airway epithelial cells reported the observation that cytoplasmic levels of AUF1 increased during infection [134]. Cytoplasmic relocalization of AUF1 was also observed following poliovirus, HRV-14, CVB3, EV71, or EMCV infection of human cells [129,130,131,132]. For two of the viruses studied, poliovirus and CVB3, AUF1 was shown to relocalize as a result of the disruption of nuclear-cytoplasmic trafficking by the viral 2A proteinase [129,131]. Given the cytoplasmic life cycle of these viruses, relocalization of AUF1 to the cytoplasm may contribute significantly to its negative impact on virus replication.

Most reports aimed at determining the mechanism by which AUF1 acts as a restriction factor point toward a negative regulation of viral IRES-driven translation. The interaction of AUF1 with the 5′ NCR of vRNA has been demonstrated for poliovirus, HRV, and EV71, and this interaction was narrowed to sites within the poliovirus and EV71 IRES [129,132]. Using in vitro translation or bicistronic reporter assays, AUF1 was shown to negatively regulate both poliovirus and EV71 translation, likely through a direct interaction with the viral IRES [129,132]. For EV71, it was shown that the negative effect of AUF1 on viral translation may be a result of competitive binding to the viral IRES with a known ITAF and AUBP, hnRNP A1 [132,135,136]. hnRNP A1 also acts as an ITAF for HRV-2, but opposing regulation by hnRNP A1 and AUF1 has not yet been investigated for this virus [137]. An additional study has suggested that AUF1 may play a role in destabilizing CVB3 RNA. It was shown that AUF1 can bind directly to the CVB3 3′ NCR and that knockdown of AUF1 leads to increased stability of a CVB3 3′ NCR reporter construct [131]. Whether AUF1 promotes the decay of genomic CVB3 RNA has not yet been determined.

Like many host restriction factors, AUF1 is cleaved during infection. For poliovirus, HRV, and CVB3, AUF1 is cleaved by the 3C (and precursor 3CD) viral proteinase [131,133]. Cleavage of AUF1 by poliovirus 3CD proteinase, at a site within the *N*-terminal dimerization domain, reduces its ability to bind the 5′ NCR, which suggests that this process may act as a virus defense against AUF1 [129]. Interestingly, during EMCV infection of mouse cells, AUF1 did not have a negative impact on virus replication and remained uncleaved throughout infection [130]. Overall, these findings support the idea that AUF1 cleavage by viral proteinases ameliorates its negative effect on virus replication. However, many of the experiments demonstrating the negative effect of AUF1 on EV replication were performed using AUF1 knockout or knockdown cell lines. While a direct association with vRNA has been shown for several EVs, there may be additional indirect contributions to the negative effect of AUF1 resulting from the dysregulation of AUF1 target mRNA and ncRNA.

Similar to AUF1, KSRP is another AUBP that has been best characterized for its role in promoting AMD of target transcripts. Additional roles in transcription, alternative splicing, and miRNA maturation have also been described for KSRP [109,138,139,140]. Like AUF1, KSRP was identified in an RNA affinity screen for its association with vRNA; in this case, as a novel EV71 5′ NCR-binding protein. KSRP was shown to relocalize from the nucleus to the cytoplasm during infection and to associate with the EV71 5′ NCR, with binding occurring at multiple sites within the viral IRES. Using protein pulse-labeling and bicistronic reporter assays, KSRP was shown to be a negative ITAF for EV71 [141,142]. Like AUF1, KSRP is cleaved during infection. However, KSRP is not a substrate for the viral 2A or 3C proteinases but is instead cleaved and degraded through the activity of caspases and the proteasome and autophagy pathways [142]. KSRP has not yet been shown to act as a negative regulator of other picornavirus infections, but it was identified as a poliovirus RNA-binding protein using TUX-MS and thus, may have a similar effect on other EVs [29].

Other AUBPs have been shown to associate with picornavirus RNAs and participate in the picornavirus replication cycle; however, these proteins are not typically linked to AMD. Instead, these AUBPs are often associated with stabilization or translation of mRNA (discussed in Section 2). hnRNP A1, the AUBP that was shown to compete with AUF1 for binding to the EV71 IRES, is a multifunctional protein involved in transcription, alternative splicing, mRNA localization, translation, and stability [143]. hnRNP A1 has been reported to destabilize mRNAs bearing AREs [144] or an ARE-like motif (a motif which was identified in ~7% of mRNAs) [145]. However, instead of destabilizing EV71 RNA, hnRNP A1 is re-purposed by the virus as a positive regulator of translation [135]. Among its many functions, hnRNP A1 has also been shown to act as an ITAF for cellular IRESs and it is this function that is utilized to promote virus replication [135,146,147].

## 7. MicroRNA-Mediated Decay

MicroRNAs (miRNAs) are small, regulatory RNAs produced in eukaryotic cells that bind to complementary sites in mRNA and act to translationally repress or signal the degradation of target transcripts [148]. Biogenesis of miRNA begins in the nucleus, where precursor miRNAs (pre-miRNAs) are cleaved from hairpin structures within primary miRNA transcripts by the RNase III nuclease, Drosha [149]. Following export to the cytoplasm, pre-miRNAs are further processed to mature miRNAs by Dicer, another RNase III nuclease [150]. Mature miRNAs are bound by a member of the Argonaute protein family within the RNA-induced silencing complex (RISC), which together act as the effector complex that targets complementary mRNAs for RNA interference [151].

miRNA binding can lead directly to mRNA degradation, or indirectly through degradation of repressed mRNAs in PBs. Direct degradation of a miRNA target in animal cells is most often initiated through recruitment of deadenylases by the RISC, and on rare occasions, by endonucleolytic cleavage of the mRNA by Argonaute 2 (Ago2), the only catalytically active member of the Argonaute family [148,152]. Until a few years ago, it was assumed that antiviral potential of the miRNA pathway is not utilized during picornavirus infections, since the cytoplasmic replication cycle of these viruses does not encounter miRNA biogenesis pathways in the nucleus. However, regions of the viral genome, such as the IRES, contain hairpin structures that resemble structured miRNA transcripts. These structured regions in the vRNA provide an opportunity for the cytoplasmic miRNA machinery to generate miRNA-like small RNAs.

Recent studies using deep sequencing techniques have revealed that viral small RNAs (vsRNAs) are produced from HAV, EMCV and EV71 RNA in a Dicer-dependent manner [153,154,155]. While the roles of the HAV and EMCV vsRNAs during infection have not been elucidated, the study of EV71 vsRNAs has revealed interesting new ways in which the virus re-purposes yet another potentially antiviral cellular pathway. One of the EV71 vsRNAs, vsRNA-1, is derived from the second stem loop (SL-II) of the 5′ NCR and negatively regulates viral translation and replication [155]. Instead of inhibiting virus replication via canonical miRNA mechanisms, it was discovered that vsRNA-1 may regulate EV71 IRES-driven translation by promoting the binding of both positive and negative ITAFs to SL-II of the 5′ NCR. Surprisingly, vsRNA-1 recruits Ago2 to the vRNA, but instead of acting as a negative regulator through translational repression or cleavage of vRNA, Ago2 acts as a positive regulator of translation [27]. These data reveal the possibility for a new and interesting regulator in the picornavirus life cycle. Whether these small, virus-derived RNAs have a negative impact on vRNA stability similar to miRNA has yet to be determined.

## 8. Concluding Remarks

Picornavirus disruption of cellular RNA decay machinery generally involves a broad approach of cleaving, degrading, inhibiting, disassembling, or re-purposing components of these processes. However, several pathways that may contribute to vRNA instability have received relatively little attention to date, and the focus has been on only a few members of the *Picornaviridae* family. For instance, 5′→3′ RNA degradation is inhibited during infection through cleavage of decapping enzymes and Xrn1, but it is unclear whether exosomes, which participate in 3′→5′ RNA degradation, remain intact. There has also been little investigation into the cellular proteins that bind vRNA and which promote vRNA stability, or conversely, promote degradation. Insight into these issues can be provided by approaches like TUX-MS, which may reveal novel RNA-binding proteins that contribute to vRNA stability. In addition, studying the picornavirus “cleavome,” or host proteins that are cleaved by viral proteinases during infection, may reveal new proteins whose functions are disrupted during infection. These cleaved proteins could include negative regulators of vRNA stability. Studies like these will reveal not only new viral mechanisms to preserve the stability of their encoded RNAs, but also host defense mechanisms that target vRNA for degradation.
